# Practitioner Acceptability of Virtual Reality Therapy in Mental Health Care: Evidence From Greece

**DOI:** 10.1002/brb3.71322

**Published:** 2026-03-30

**Authors:** Rigina Skeva, Nektaria Pedioti, Maria Eleni Efthymiou, Vaia Fotou, Lynsey Gregg, Maria Papadakaki

**Affiliations:** ^1^ Department of Computer Science, Faculty of Science and Engineering University of Manchester Manchester UK; ^2^ Department of Social Work, School of Health Sciences Hellenic Mediterranean University Crete Greece; ^3^ Division of Psychology and Mental Health, Faculty of Biology, Medicine and Health University of Manchester Manchester UK

**Keywords:** acceptability, digital health, mental health, virtual reality therapy (VRT)

## Abstract

**Introduction:**

Mental health conditions represent a global public health challenge, with persistent treatment gaps across healthcare systems despite available, effective interventions. Virtual reality therapy (VRT) offers a promising avenue for improving mental health service delivery; however, understanding practitioner acceptability is essential for successful implementation.

**Methods:**

This cross‐sectional study examined the acceptability of VRT among mental health practitioners in Greece. Forty professionals (psychologists, psychiatrists, nurses, and social workers) working across hospital, community, and long‐term care settings completed a structured questionnaire assessing familiarity with virtual reality and VRT, and the acceptability of VRT for anxiety disorders, bipolar disorder, schizophrenia, and its clinical integration.

**Results:**

Overall, VRT was perceived as acceptable; acceptability was highest for anxiety disorders, followed by bipolar disorder and schizophrenia. Acceptability of VRT's clinical integration was low, with participants expressing concerns regarding service readiness, practitioner training, and long‐term outcomes. Although practitioner familiarity with VRT was limited, they were open to VRT use, particularly as an adjunct.

**Conclusions:**

To our knowledge, this is the first study to examine VRT acceptability among mental health practitioners in Greece. The findings indicate that acceptability is shaped more by training, infrastructure, and culturally sensitive implementation than by resistance to technological innovation.

## Graphical abstract

In a survey of 40 Greek mental health practitioners, virtual reality therapy (VRT) was viewed as acceptable—particularly for anxiety disorders and as an adjunct for bipolar disorder and schizophrenia—yet concerns about training, infrastructure, and service readiness limited support for routine clinical integration.

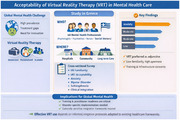

## 1 Introduction

Mental health conditions represent a significant and growing public health concern worldwide. It is estimated that one in seven people globally has a mental health disorder (World Health Organization 2025). Anxiety disorders are the most frequent (∼359 million), followed by bipolar disorder, depression, and schizophrenia (World Health Organization 2025). In Greece, one in five people experiences a mental health disorder, placing the country among the most affected in Europe (Organisation for Economic Co‐operation and Development & European Observatory on Health Systems and Policies 2023). Although effective treatment and prevention interventions exist, global statistics reveal that health systems remain under‐resourced and struggle to deliver targeted care for most people, even in developed countries (World Health Organization 2025).

A key priority of the WHO and European Union is to promote access to mental health treatment in social and health care services, through integrated, innovative, and scalable strategies, as described in the WHO's Comprehensive Mental Health Action Plan 2013–2030 (World Health Organization 2021) and the EU Commission's Comprehensive Approach (European Commission 2023). Greece, in collaboration with WHO, is actively reforming mental health services to meet the Action Plan's objectives (World Health Organization 2023) and has specifically developed a dedicated strategy for the digital transformation of its healthcare ecosystem (Hellenic Republic n.d.), supported by its National Recovery and Resilience Plan (Greece 2.0 n.d.).

Within this context, digital tools, such as virtual reality therapies (VRT), represent promising candidates due to their potential to offer novel, personalized, and accessible care to large parts of the population, especially in remote and rural areas (Pedram and Piatkowski 2025; Ong et al. 2024; Aristawati et al. 2025). This is particularly relevant for Greece, where the geographic dispersion of multiple remote islands and limited mainland connectivity—especially during winter—contributes to significant inequalities in healthcare access (Kassas et al. 2023). Socioeconomic barriers, including low levels of health literacy and an increased risk of poverty affecting approximately one‐third of the population, significantly limit access to healthcare (Kyriopoulos et al. 2025). In parallel, demographic factors, such as a large aging population with complex, personalized care needs and under‐resourced healthcare units, highlight the need for novel and scalable solutions to improve access to personalized healthcare (Kyriopoulos et al. 2025).

VRT has already been evaluated in clinical, mental health treatment contexts (Zeng et al. 2025; Emmelkamp and Meyerbröker 2021). Its use as an immersive, exposure method, delivered as a stand‐alone intervention or combined with available treatment schemes, yielded promising results (Zeng et al. 2025; Cushnan et al. 2025; Emmelkamp and Meyerbröker 2021; Freeman et al. 2017; Maples‐Keller et al. 2017; Valmaggia et al. 2016). In essence, VRT applications can recreate high‐risk, anxiety‐related situations within interactive virtual environments, including simulated social encounters with virtual characters. Exposure can be controlled by the practitioner and the patient, be tailored to their therapeutic needs, and delivered remotely. This transforms VRT into a practical, accessible, and engaging tool for assessment, training, and treatment purposes (Bell et al. 2024; Freeman et al. 2017).

However, VRT's institutional adoption within public health systems remains limited, highlighting the need for systematic assessment of implementation requirements, primarily relating to treatment acceptability and practitioner readiness, and formal delivery protocols (Pedram and Piatkowski 2025; Bell et al. 2024; Ong et al. 2024; Chung et al. 2022; Park et al. 2019). A better understanding of practitioner views of VRT would assist in developing robust and informed integration frameworks.

Previous studies have focused on the perceptions of practitioners on VRT for psychological treatment in general (Segal et al. 2011) and substance use disorders (SUDs) (Skeva et al. 2021; Arissen et al. 2023). VRT was perceived as beneficial for psychological and SUD treatment, mainly due to the practicality it offers in cue exposure; this was strongly endorsed by cognitive behavioral therapists compared to psychodynamic ones, which is justifiable given that exposure is often utilized in their practice (Skeva et al. 2021; Arissen et al. 2023).

Implementation costs (e.g., hardware, software development and maintenance, technical support) and training (including being offered a detailed manual) were identified as the main challenges of VRT (Skeva et al. 2021; Segal et al. 2011). Familiarity with virtual reality (VR) and VRT has been associated with greater treatment acceptability (Skeva et al. 2021; Segal et al. 2011), while positive attitudes appear to increase following clinical use (Arissen et al. 2023). Reported benefits after clinical use of VRT included enhanced role‐play possibilities, increased variability in therapeutic activities, and greater engagement during sessions, while dedicated technical support, training, and user guidance were also viewed as helpful (Arissen et al. 2023).

The evidence base on VRT's acceptability, however, is limited to the views of psychologists and therapists, excluding frontline professionals such as social workers, nurses, and psychiatrists, that typically deliver mental health interventions (Arissen et al. 2023; Skeva et al. 2021; Segal et al. 2011). VRT's acceptability for use with prevailing mental health disorders, such as bipolar disorder, anxiety disorders, and schizophrenia, has not also been assessed. Finally, the perceptions, familiarity, and readiness of practitioners in Greece about VRT in mental health remain entirely unexplored, even though VRT could help improve access disparities in healthcare, caused by Greece's geographical structure and socioeconomic burdens (Kassas et al. 2023; Kyriopoulos et al. 2025).

This study is part of a larger project exploring the acceptability of VRT in the Greek mental health services. The primary aim of this study was to explore the acceptability of VRT in Greek mental health services specifically for the treatment of bipolar disorder, anxiety disorders, and schizophrenia, and the readiness of Greek practitioners and mental health services for VRT's clinical integration. Due to scope and feasibility constraints, depression was excluded to maintain analytical focus. Social workers, nurses, psychologists, and psychiatrists were surveyed to bridge the relevant representation gap in the literature and capture their views and familiarity with VRT for the treatment of disorders that impact the largest part of the population worldwide and in Greece (World Health Organization 2025; Organisation for Economic Co‐operation and Development & European Observatory on Health Systems and Policies 2023).

## 2 Materials and Methods

### 2.1 Sample and Recruitment

Participants were recruited via purposive and convenience sampling. The inclusion criteria for participating in the online survey were (1) to be 18 years old or older, (2) to work as a practitioner delivering mental health treatments at least 1 year, and (3) to be employed by an official, public, or private social care or mental health service of the Greek, public healthcare system. Recruitment took place by inviting personnel working in these services in the region of Crete, Greece, through announcements disseminated via their institutional email newsletters. The advertising text asked for voluntary participation in a survey exploring the views of mental health practitioners in the Greek healthcare system about the potential integration of VRT into their practice. Of the potential participants, 41 provided consent to complete the study, of which one withdrew at some point of the survey, and 40 participants completed the whole survey, from 14 different mental healthcare institutions.

### 2.2 Procedure

Data collection was conducted online through the Google Forms platform. A link was provided that led directly to the survey, participant information sheet, and consent form. No compensation was offered for taking part. After reviewing the online information sheet, participants confirmed eligibility and provided consent. Then they were presented with an explanation of VRT, of the VR equipment and set‐up that are typically used in clinical practice, and of its effectiveness so far as per recent literature. The clinical application and effectiveness of VRT were presented for each type of disorder. They were asked to complete the survey in one session, and the platform required responses to all items before progressing. The questionnaire was designed to be user‐friendly, requiring no more than 15–20 min to complete, with three, thematically coherent sections. Participation was completely anonymous, and no personal or identifying information (e.g., name, email, address) was collected, thereby ensuring full protection of personal data.

### 2.3 Measures

#### 2.3.1 Demographic Characteristics (Section 1)

Participants were asked to provide demographic information, namely the age group they belong to, occupation, mental health facility type they work in, and range of years of professional experience.

#### 2.3.2 Familiarity With VR (Section 2)

Participants were next asked about their familiarity with VR. Three 5‐point Likert‐type items (1 = not at all familiar to 5 = very familiar) assessed participants’ personal usage of VR, overall familiarity with VR technologies, and familiarity with the clinical use of VR. Familiarity with VR and VRT were considered, as they have been linked to increased treatment acceptability (Skeva et al. 2021; Segal et al. 2011). To facilitate analysis, the three Likert‐type items assessing participants’ familiarity with VR were combined into a single composite score—producing a scale approximating continuous data as per standard practice (Central Limit Theorem—Field 2018). Internal consistency was high (Cronbach's *α* = 0.851), indicating that the items measured the same underlying construct and could be treated as a single variable.

#### 2.3.3 Acceptability of VRT (Section 3)

In the final section, the views of participants on the use of VR in the treatment of three mental health disorders (anxiety disorders, bipolar disorder, schizophrenia), and on its clinical integration as a therapeutic tool were evaluated. Nineteen items were developed for this study to examine perceived effectiveness of VRT per disorder type, its potential to enhance existing treatment approaches, and its future therapeutic prospects.

Questions for each disorder type explored whether VRT could help manage symptoms, exacerbate symptoms, and its long‐term clinical suitability. Some questions were specifically tailored for each disorder type to address the suitability of VRT to be combined with current treatment methods (e.g., exposure therapy for anxiety disorders, cognitive behavioral therapy [CBT] for bipolar disorder, or pharmacotherapy for schizophrenia). Participants were also asked about VRT's integration into clinical practice and the feasibility of implementation within mental health structures, such as easiness to be integrated into current clinical practice or whether it would be beneficial in the long term for mental health services. All items in this section were rated on 5‐point Likert scales ranging from 1 (“strongly disagree”) to 5 (“strongly agree”).

Items were combined into a single composite score for each mental health disorder (anxiety disorders, bipolar disorder, schizophrenia) as well as for VRT acceptability of its clinical integration, resulting in four distinct variables. Reliability analyses indicated good internal consistency for all scales: VRT acceptability in anxiety disorders (Cronbach's *α* = 0.774), bipolar disorder (*α* = 0.725), schizophrenia (*α* = 0.873), and overall VRT acceptability (*α* = 0.826).

### 2.4 Data Analysis

Data were analyzed in SPSS, version 29.

Descriptive statistics were employed for the analysis of demographic data and items assessing familiarity with VR and VRT. For the acceptability of VRT, mean scores were calculated for each composite variable to facilitate subsequent analyses.

Spearman's correlations were used to examine associations between familiarity with VR and VRT acceptability for each mental health disorder, as these variables violated assumptions of normality. Pearson's correlation was used to explore the relationship between familiarity with VR and VRT acceptability for clinical integration, as both variables were normally distributed.

Relationships between demographic variables and VRT acceptability were explored across disorder‐specific and clinical integration scores. Normality was assessed using Shapiro–Wilk tests. Group comparisons across age, occupation, mental health facility type, and years of professional experience were conducted using Kruskal–Wallis tests or one‐way between‐subjects ANOVAs, depending on the distribution of the data. Statistical significance was set at *p* < 0.05.

### 2.5 Ethical Approval

Ethical approval for the study was granted by the Hellenic Mediterranean University Research Ethics Committee (Ref.: 130901).

## 3 Results

### 3.1 Characteristics of Participants

A total of 40 participants completed the survey. An overview of the characteristics of participants is presented in Table 1.

**TABLE 1:** Demographic characteristics of participants.

Demographic variable	*N* (%)
	
**Age**	
25–35	10 (25%)
36–45	11 (27.5%)
45–55	15 (37.5%)
56+	4 (10%)
**Occupation**	
Social worker	13 (32.5%)
Nurse	14 (35%)
Psychiatrist	2 (5%)
Psychologist	11 (27.5%)
**Mental health facility type**	
Hospitals	10 (25%)
Outpatient and community services	13 (32.5%)
Long‐term care or rehabilitation units	17 (42.5%)
**Years of professional experience**	
1–5	12 (30%)
6–10	3 (7.5%)
11–15	4 (10%)
15+	21 (52.5%)

### 3.2 Familiarity With VR

The majority of participants (26, 65%) has never used VR before, while 12 of them (30%) reported brief use and only 2 (5%) reported regular use. In line with this, 25 participants (62.5%) stated that they were entirely unfamiliar with VR, 10 (25%) a little familiar with VR, and 5 (12.5%) very familiar with VR. Four out of five participants (32, 80%) had no knowledge about the use of VR in clinical practice, while seven (17.5%) reported having a little knowledge, and only one (2.5%) considered themselves to have good knowledge.

Overall, the mean score of the familiarity composite variable was generally low (*M* = 1.45, range = 1–3, SD = 0.63), denoting that practitioners on average did not have good knowledge of VR and VRT, including its clinical potential.

### 3.3 Acceptability of VRT

Mean scores indicated a generally positive attitude toward VRT, with higher values reflecting greater perceived acceptability. The mean scores of VRT's acceptability for the treatment of specific mental health disorders (anxiety disorders, bipolar disorder, schizophrenia) and as an intervention method to be clinically integrated are presented in Table 2.

**TABLE 2:** VRT acceptability mean scores per disorder type and for clinical integration.

VRT acceptability	Minimum	Maximum	Mean	Std. deviation
Anxiety disorders	1.00	4.60	3.56	0.63
Bipolar disorder	1.00	3.8	3.20	0.55
Schizophrenia	1.00	4.00	3.00	0.80
Clinical integration	1.00	4.00	2.87	0.63

VRT was considered most acceptable in the treatment of anxiety disorders, whereas the lowest acceptability was reported when its clinical integration was examined.

#### 3.3.1 VRT Acceptability in Anxiety Disorders

Participants expressed relatively high agreement that VR could be effective in managing anxiety symptoms (*M* = 3.70, SD = 0.91) and in supporting gradual exposure to anxiety‐provoking situations, consistent with exposure therapy approaches (*M* = 3.78, SD = 0.89).

In addition, participants reported strong agreement regarding the potential for VR to be effectively combined with other therapeutic methods, such as CBT (*M* = 3.90, SD = 0.87), and viewed VR as having significant future potential in the treatment of anxiety disorders (*M* = 3.73, SD = 0.91). In contrast, respondents tended to moderately disagree that the use of VR could intensify anxiety symptoms (*M* = 2.70, SD = 0.79).

#### 3.3.2 VRT Acceptability in Bipolar Disorder

Participants moderately agreed that VR could improve emotional stability in patients with bipolar disorder (*M* = 3.10, SD = 0.78) and enhance patients’ ability to recognize early signs of manic or depressive episodes (*M* = 3.23, SD = 0.73). Higher levels of agreement were observed regarding the potential for VR to be effectively combined with existing therapeutic models, such as CBT (*M* = 3.53, SD = 0.85), as well as its future potential as a therapeutic tool for bipolar disorder (*M* = 3.33, SD = 0.83). participants were neutral on whether the use of VR, participants tended to nor disagree or agree that the use of VR could trigger or exacerbate symptoms of bipolar disorder (*M* = 2.83, SD = 0.81).

#### 3.3.3 VRT Acceptability in Schizophrenia

Participants revealed more cautious and moderate attitudes toward the application of VR in this clinical population. They tended to nor disagree or agree that VR practice could lead to remission of psychotic symptoms (*M* = 2.70, SD = 0.88).

Moderate levels of agreement were observed regarding the potential for VR to be used in combination with pharmacotherapy to improve symptom management (*M* = 3.30, SD = 0.94). Participants also reported neutral to moderate agreement that VR could contribute to the reduction of negative symptoms of schizophrenia, such as social withdrawal and reduced mobility (*M* = 3.00, SD = 0.85).

#### 3.3.4 VRT Acceptability in Clinical Integration

Overall, participants expressed moderate attitudes toward the integration of VRT into mental health services. Nor disagreement or agreement was reported regarding the perception that VR could offer patients a greater sense of control and engagement compared to other therapeutic methods (*M* = 2.83, SD = 0.88), as well as for the general opinion on the inclusion of VR as a treatment method (*M* = 3.44, SD = 1.00).

Participants reported lower levels of agreement concerning the readiness of local mental health services in Greece to adopt VR‐based interventions (*M* = 2.33, SD = 0.93) and the ease of integrating VR into everyday clinical practice (*M* = 2.33, SD = 0.96). There was clear disagreement with the notion that VR could replace other therapeutic methods (*M* = 2.11, SD = 0.82).

Finally, respondents expressed moderate agreement that the implementation of VR could lead to long‐term positive outcomes in mental health services (*M* = 3.47, SD = 0.81) and viewed the overall potential for integrating VR as a therapeutic method in mental health structures as moderate (*M* = 3.28, SD = 0.97).

#### 3.3.5 Variables of VRT Acceptability

Exploratory analyses were conducted to examine whether demographic variables were associated with VRT acceptability across disorder‐specific and clinical integration acceptability scores. No statistically significant differences or associations were found for age, occupation, mental health facility type, years of professional experience, and familiarity with VR across any of the VRT acceptability measures (all *p* > 0.05).

## 4 Discussion

This study examined the acceptability of VRT in mental health practitioners in Greece, focusing primarily on its usability in anxiety disorders, bipolar disorder, schizophrenia and on its potential integration in current mental health services. The acceptability of VRT in the treatment of these specific disorders has not been assessed before, nor were the views of frontline mental health professionals such as social workers, nurses, and psychiatrists. The acceptability of VRT for integration into the Greek mental health services was also unexplored before this study.

Overall, VRT was perceived as an acceptable intervention for the treatment of mental health disorders, with mean acceptability scores at or above the midpoint of the scale (≥ 3 on a 5‐point Likert scale). The highest acceptability of VRT was reported for anxiety disorders, followed by bipolar disorder, and then schizophrenia. Clinical integration acceptability demonstrated the lowest mean score. The highest acceptability to be reported in anxiety disorders aligns with its extensive application in this domain, due to its ability to offer controlled, immersive exposure (VR exposure therapy [VRET]) and increased practicality compared to traditional exposure techniques—in vivo and imaginal exposure therapies (Baghaei et al. 2021; Maples‐Keller et al. 2017; Carl et al. 2019; Freeman et al. 2017). Strong compatibility of VRT with current exposure models and CBT could lead to increased familiarity among practitioners in this domain and influence acceptability. Familiarity has been acknowledged as a predictor of VRT's acceptability in relevant literature (Skeva et al. 2021; Segal et al. 2011; Tarrier et al. 2006).

Acceptability regarding bipolar disorder was lower compared to anxiety disorders, with participants seeing VRT as more acceptable when suggested as an adjunct to traditional treatments (such as CBT) rather than a stand‐alone intervention (as opposed to VRET). The evidence base for VRT with bipolar disorder remains limited but promising, supporting its assistive role in treatment (de Pablo et al. 2024; Rus‐Calafell et al. 2018) and aligning with the moderate optimism of participants in this study about its clinical potential.

Similarly, for schizophrenia, participants endorsed the use of VRT complementary to pharmacotherapy and its potential to address negative symptoms such as social withdrawal. This aligns with earlier research that highlights the adjunctive role of VR as a psychosocial intervention in psychosis, evidently assisting in social skills training, cognitive remediation, and paranoia reduction (de Pablo et al. 2024; Freeman et al. 2023; Rus‐Calafell et al. 2018; Valmaggia et al. 2016). VRT being viewed more favorably as a complementary tool integrated into existing therapeutic frameworks, rather than stand‐alone, was also underscored as a clinical requirement of its use in practitioner acceptability studies about SUDs (Skeva et al. 2021).

The lowest score was reported for the acceptability of VRT regarding its clinical integration. This could be attributed to the fact that sub‐items of this scale focused on the ease of integration into clinical practice and the readiness of mental health practitioners—complex aspects identified as challenges in prior studies (Skeva et al. 2021; Segal et al. 2011). Moderate scores in sub‐items about the long‐term benefits of incorporating VRT into mental health services and expected effectiveness over traditional treatment methods may also be linked to limited familiarity with VRT in the sample. Previous studies have suggested that practitioners’ perspectives over these topics change after VRT's clinical use, showcasing increased acceptance (Arissen et al. 2023). This may also be attributed to the lack of robust legal and organizational pathways for integrating digital tools in the Greek healthcare system (Voutsidou 2022). Limited familiarity with VRT and its clinical use, alongside perceived challenges in service integration, highlight the importance of practitioner training and structural changes prior to the implementation of VRT in the Greek healthcare system and worldwide.

### 4.1 Limitations

This study has several limitations that should be considered when interpreting the findings. First, the sample was small (*n* = 40) and geographically restricted to Crete, which limits statistical power and generalizability. The lack of significant predictors could be attributed to limited power rather than true null effects. In addition, psychiatrists were underrepresented (*n* = 2), which potentially led to medical perspectives to not being adequately reflected. A broader national and diverse sample would allow for more generalizable conclusions regarding the acceptability of VRT.

Second, the use of convenience sampling via institutional email newsletters introduces the possibility of self‐selection bias. Practitioners who are more interested in innovation or digital technologies may have been more likely to participate, potentially increasing acceptability estimates.

Third, most participants had not employed VR in their clinical practice, so their views were speculative. Prior research with practitioners suggests that hands‐on exposure leads to higher acceptability ratings; therefore, the current conclusions should be considered preliminary.

Fourth, although the questionnaire showed satisfactory internal consistency, it has not been externally validated. Utilizing validated implementation approaches (such as the Unified Theory of Acceptance and Use of Technology [UTAUT], Consolidated Framework for Implementation Research [CFIR], or Non‐adoption, Abandonment, Scale‐up, Spread, and Sustainability [NASSS]) could have enhanced construct validity and improved comparability with prior research.

Finally, the absence of qualitative data limits deeper understanding of perceived barriers and facilitators, such as costs, workflow implications, ethical concerns, and liability issues. Incorporating interviews or open‐ended responses would have enriched interpretation and strengthened the study's contribution to the digital mental health literature.

### 4.2 Strengths

Despite these limitations, this is the first study to explore the acceptability of VRT in mental health treatment in Greece. It is also the first acceptability study with a specific focus on individual disorders, namely anxiety disorders, bipolar disorder, and schizophrenia. In this survey, a wide range of mental health practitioners took part, including psychologists, therapists, social workers, nurses, and psychiatrists, allowing diverse perceptions of key frontline professionals to emerge—bridging the relevant gap in literature. Participants were employed by a variety of mental health services, including hospitals, outpatient and community services, and long‐term care or rehabilitation units. This ensured that identified challenges, benefits, and VRT acceptability linking to its clinical integration would be relevant to all, potential mental health facility types—which was also not explored before in previous research.

Future research should expand on the present findings through recruitment of a larger, national sample, especially those underrepresented in this study, such as psychiatrists. More balanced distribution across practitioner roles could allow group comparisons in VRT acceptability scores, offering valuable insights about individual perspectives, and structural and educational needs linking to each role. Acceptability of VRT for the treatment of other disorder types, such as depression and SUDs, and of cases where comorbidity of disorders exists, should also be considered in future studies, to holistically inform VRT integration in Greek mental health services. Mixed‐method designs, including in‐depth questioning of practitioners depending on survey responses, could further enrich acceptability outcomes.

## 5 Conclusion

This study demonstrates generally positive, yet disorder‐specific, acceptability of VRT among mental health practitioners in Greece. Acceptability was highest for anxiety disorders, followed by bipolar disorder and schizophrenia. However, acceptability was low for clinical integration. For anxiety disorders, VRT's use as a complementary, exposure method was endorsed, while in bipolar disorder and schizophrenia VRT was seen suitable as adjunctive to traditional treatments in general (not only for exposure purposes) and mainly as a psychosocial tool. Despite limited familiarity with VRT and minimal prior clinical exposure, practitioners showcased openness toward its future use, highlighting that acceptability is influenced more by structural and educational barriers than by resistance to technological innovation.

From a global mental health perspective, these findings underscore the importance of practitioner training, service readiness, and culturally sensitive implementation strategies in the successful adoption of immersive digital interventions. Successful implementation of VRT in mental health services requires disorder‐specific approaches and clear positioning of VR as an assistive rather than substitutive intervention, especially in complex psychiatric conditions; informed integration protocols, as shaped by acceptability studies run throughout VRT's implementation lifecycle, can lead to enhanced, innovative mental health care across diverse healthcare systems.

## Author Contributions

R.S. contributed to conceptualization, led the methodology, conducted the formal analysis, and wrote the original draft, with overall supervision and review and editing of the manuscript. N.P. conceived the study, methodology, project administration, supervision, and visualization. M.E.E. and V.F. conducted the investigation, data curation, and formal analysis. L.G. contributed to validation, supervision, and manuscript review and editing. M.P. contributed to supervision, validation, and project administration.

## Funding

The authors have nothing to report.

## Ethics Statement

Ethical approval for the study was granted by the Hellenic Mediterranean University Research Ethics Committee (Ref.: 130901). The research conforms to the provisions of the Declaration of Helsinki. All persons gave their informed consent prior to their inclusion in the study.

## Conflicts of Interest

The authors declare no conflicts of interest.

## Data Availability

The data that support the findings of this study are available from the corresponding author upon reasonable request.
